# Should DNA sequence be incorporated with other taxonomical data for routine identifying of plant species?

**DOI:** 10.1186/s12906-017-1937-3

**Published:** 2017-08-31

**Authors:** Tanakorn Suesatpanit, Kitisak Osathanunkul, Panagiotis Madesis, Maslin Osathanunkul

**Affiliations:** 1 Department of Biology, Faculty of Science, Chiang Mai University, Chiang Mai, 50200 Thailand; 2 Department of Computer Science, Faculty of Science, Maejo University, Chiang Mai, 50290 Thailand; 3 Institute of Applied Biosciences, Centre for Research & Technology Hellas (CERTH), Thessaloniki, Greece; 4 Center of Excellence in Bioresources for Agriculture, Industry and Medicine, Chiang Mai University, Chiang Mai, Thailand

**Keywords:** DNA barcoding, High Resolution Melting, Medicinal plants, Raw material, Molecular data

## Abstract

**Background:**

A variety of plants in Acanthaceae have long been used in traditional Thai ailment and commercialised with significant economic value. Nowadays medicinal plants are sold in processed forms and thus morphological authentication is almost impossible. Full identification requires comparison of the specimen with some authoritative sources, such as a full and accurate description and verification of the species deposited in herbarium. Intake of wrong herbals can cause adverse effects. Identification of both raw materials and end products is therefore needed.

**Methods:**

Here, the potential of a DNA-based identification method, called Bar-HRM (DNA barcoding coupled with High Resolution Melting analysis), in raw material species identification is investigated. DNA barcode sequences from five regions (*matK*, *rbcL*, *trnH-psbA spacer region*, *trnL* and ITS2) of Acanthaceae species were retrieved for in silico analysis. Then the specific primer pairs were used in HRM assay to generate unique melting profiles for each plants species.

**Results:**

The method allows identification of samples lacking necessary morphological parts. In silico analyses of all five selected regions suggested that ITS2 is the most suitable marker for Bar-HRM in this study. The HRM analysis on dried samples of 16 Acanthaceae medicinal species was then performed using primer pair derived from ITS2 region. 100% discrimination of the tested samples at both genus and species level was observed. However, two samples documented as *Clinacanthus nutans* and *Clinacanthus siamensis* were recognised as the same species from the HRM analysis. Further investigation reveals that *C. siamensis* is now accepted as *C. nutans*.

**Conclusions:**

The results here proved that Bar-HRM is a promising technique in species identification of the studied medicinal plants in Acanthaceae. In addition, molecular biological data is currently used in plant taxonomy and increasingly popular in recent years. Here, DNA barcode sequence data should be incorporated with morphological characters in the species identification.

## Background

Herbal drugs have been used since ancient times for the treatment of a range of ailments. Medicinal plants have played a key role in world health. Around 80% of world population used traditional medicine for their primary healthcare owing to its low cost and people’s faith [1]. Medicinal plants are distributed throughout the world, but they are most abundant in tropical countries. Over the past decades, interest in herbal medicine has increased dramatically not only in developing countries but also in industrialised countries. In Thailand, a variety of plants in the family Acanthaceae have long been used as key ingredients in traditional medicines and being used in the treatment of various ailments. Six species were included in National List of Essential Medicine of Thailand. Some of the most recalled and economically significant species are *Acanthus ebracteatus* (Ngueg-Pla-Mor), *Andrographis paniculata* (Fah-TaLai-Jone), *Clinacanthus siamensis* (Sa-Led-Pang-Porn), *Thunbergia laurifolia* (Rang-Chuet). The popularity of traditional medicines might be reflected from their availability in household drug cabinet and both traditional and modern drug stores. The latest value of Thai herbal products manufactured by the government as estimated in 2010 by Thai Health organisation was ca. 8000 billion baht (300 million USD), excluding countless private suppliers. The economic importance of the herbal supplement industry is increasing every year. As the herbal industry grows, consumer safety is one issue that cannot be overlooked. Most of herbal raw material used in the production of herbal medicines is procured from wild sources. The manufacturers of these herbal medicines should be subject to strict controls regarding each product’s quality and ingredients. Routine testing or identifying of raw materials should be performed to ensure that the raw materials used in pharmaceutical products are suitable for their intended use. This is because many medicinal plants have similar macro-structural morphology among species within the same genus, whereas others are under-differentiated using vernacular names (i.e. where the same name is applied to multiple species within the same genus). Relying solely on morphological characters or vernacular names can lead to confusion in species identification [2], and subsequent substitution, either accidental or intentional, during the manufacturing process. Misidentification of the constituent plants may lead to the inclusion of undesirable, unrelated species, with a potential health risk to the end users. Substitution of the product’s ingredients either intentionally or inadvertently can have negative effect on both consumers and producers. The development and application of reliable methods for species identification of herbal raw materials and their derived products is critical for the enforcement of good manufacturing practice and to avoid safety and efficacy issues.

Molecular identification through DNA barcoding is a powerful method for the identification of both animal and plant species. DNA barcode is a short standardised DNA region(s) used to identify organisms as specific as species level. Proposed in 2003 by Hebert, this concept has been proved successful in identification of various groups of animal e.g. bird, reptile, insect and mammal using a mitochondrial gene *cytochrome oxidase* I (COI). [3–5]. In plants, however, it is more complicated. The low substitution rate of COI in land plants necessitated the search for the other plant DNA barcodes. While different markers performed best in different groups of plants and each possessed different strengths and weaknesses, as a result of efforts from many research groups, various DNA regions (e.g. *mat*K, *rbc*L, *trnH-psbA spacer region*, *trn*L, ITS) were proposed for plant barcoding. [6–13]. Accordingly, massive data of DNA barcodes in wide range of organisms, accumulated year by year despite its short history, is presently available on online databases readily to be utilised.

In developing countries including Thailand where sequencing facilities may not be widely equipped in laboratories, molecular studies that require sequencing service from outsources such as genotyping and DNA barcoding could have a number complications ranging from time-consuming, economical inefficiency, limitation of routine operation, etc. High resolution melting (HRM) is an emerging method for monitoring DNA dissociation (“melting”) kinetics, and through this methods, even a single base change between samples can be readily detected and identified [14–16]. HRM involves tracking of change in light intensity emitted from double-stranded DNA intercalating fluorescent dye as the temperature gradually increases in ‘high resolution’ increment of at most 0.2 °C or lower to denature DNA fragment [17]. The denaturation thermodynamics of individual double-stranded DNA to single strands are based on the binding affinities of individual nucleotide pairs, and melting pattern will vary due to variations in product sizes, GC contents and nucleotide composition. These differences are inferred in terms of varying melting temperatures (T_m_). Fluorescent measurements are collected at standard temperature increments and plotted as a “melting curve”. The curve’s shape and peak are characteristic for each sample, allowing for comparison and discrimination among samples. By combining DNA barcode with HRM (called Bar-HRM), limitation of DNA barcoding technique could be minimized as the sequencing is not required for Bar-HRM. Bar-HRM was proved to be a powerful tool for species identification that is capable not only to identify but also to quantitatively detect adulterants. The power of Bar-HRM have been demonstrated in bean crops [18], Hazel nut [19], Berry [20], and recently authenticate medicinal plant species in herbal products [21–25]. The method has been only used to detect or identify adulteration or inferior quality in finished products. However, to ensure the plant products compliance with stringent quality and safety standards, identifying the raw herb at an early stage – when acquiring raw materials could help in distinguishing the substitute or adulterant. Several studies on herbal probucts showed that to one kind of herbal product, any difference in raw materials can lead to variant efficacy [26]. Few techniques have been used to distinguish the raw herb from the substitute or adulterant such as capillary electrophoresis [27], High Performance Thin layer chromatography (HPTL) [28] and microscopic examination. In addition, quality of herbal medicinal product must meet the standards. Quality applies from the field to finished Product. Good Agricultural and Collection Practice (GACP) and Good Manufacturing Practice (GMP) are examples of some of the required practices and systems which need to be followed. Herbal products must contain the correct ingredients of acceptable quality, free from unacceptable contamination, etc. Aim of this study is to evaluate the Bar-HRM technique in identifying raw herb materials to shed a new way in ensuring quality control of herbal medicine products sold in markets. In order to achieve our aim 1) the popular candidate regions including ITS2, *matK*, *rbcL, trnH-psbA spacer region* and *trnL* were analysed to find the most suitable marker for identifying medicinal plants in family Acanthaceae by Bar-HRM. 2) the effectiveness of developed Bar-HRM technique with our choice of primer pair was performed to discriminate the tested medicinal plants species. The success of this study will highlight the potential of Bar-HRM as a rapid, sensitive, economical, high-throughput and taxonomical expertise-free technique for routine identifying of herbal raw materials as quality assurance approach.

Several types of taxonomic data are being used for delimitation and description taxa and was firstly introduced as integrative taxonomy in 2005 [29, 30]. In light of the difficulty of achieving accurate morphological identification of some organism groups, molecular analyses have been shown to be valuable tool. Recently, an integrative morpho-molecular classification is gaining popularity. The DNA and morphology is the most combination used in integrative taxonomy as can be seen from scientific articles published with keywords or the words contain in the title as integrative taxonomy during 2006 to 2013 [31]. Although debate remains about the criteria to use multidisciplinary for species delimitation, it is suggested that the integrative taxonomy does not replace traditional taxonomy but it could be used to increase rigour in systematic studies [32].

## Methods

### In silico analysis of the most suitable DNA barcode

**Fig. 1:**
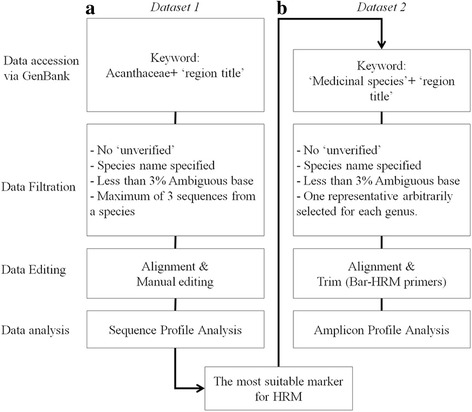
Workflow of data accession and in silico analysis to address the most suitable DNA barcode for Bar-HRM of medicinal Acanthaceae. **a**. *Dataset 1*, DNA barcodes retrieved from GenBank of the entire family Acanthaceae were included. **b**. *Dataset 2*, the best performed barcode of only medicinal species of Acanthaceae were included in analysis

**Fig. 2:**
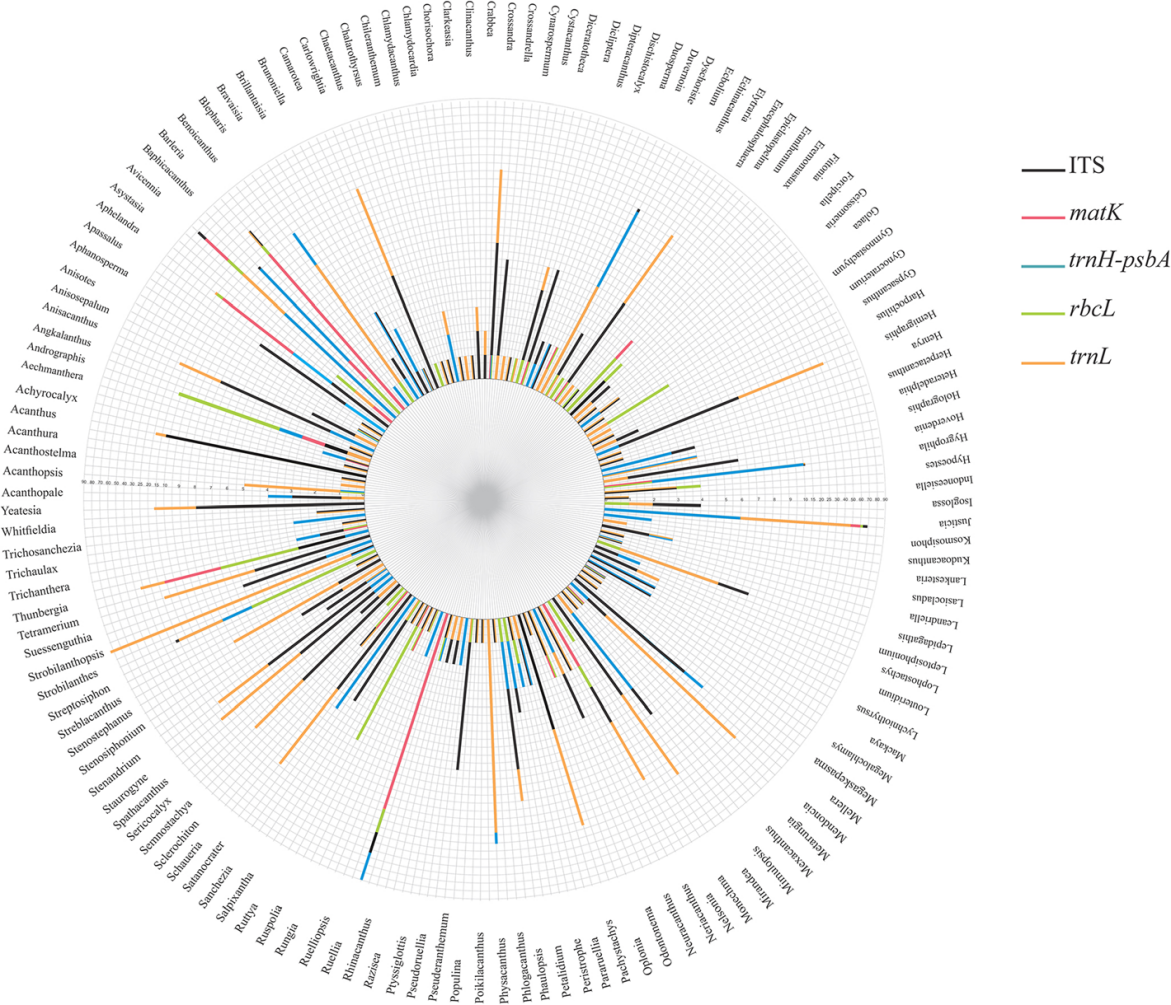
DNA sequences of Acanthaceae plant species from five chloroplast regions (*matK*, *trnH-psbA spacer region*, *rbcL*, *trnL* and ITS2) found in GenBank. In total of 2143 sequences from 140 genus were retrieved

To address the most suitable markers for identification of medicinal plants in Acanthaceae based on Bar-HRM technique, a couple datasets were constructed to conduct the sequence profile analysis (Fig. 1). The first dataset was to evaluate the sequence profile of ITS, *matK*, *rbcL, trnH-psbA spacer region* and *trnL* retrieved from GenBank which included sequences from the entire family of Acanthaceae (in total of 2143 sequences from 140 genus) (Fig. 2). On second dataset, we deepened our scope to evaluate the sequence profile of amplicon generated by the Bar-HRM primers with only medicinal plant species in Acanthaceae.

#### Dataset 1 Sequence profile of DNA barcodes of Acanthaceae

Sequences of ITS2*, matK*, *trnH-psbA spacer region*, *rbcL* and *trnL* were extracted from GenBank using keyword ‘Acanthaceae + (region title)’ on 18 February 2015. Generally, sequences obtained from public databases, including GenBank, are of low quality with no known associated herbarium vouchers. For this reason, all of the sequences were subjected to critical evaluation and any low-quality sequences were removed. Criteria used to filter the sequences were (1) sequences are not ‘unverified’ without a species name (2) contain <3% ambiguous base ‘N’ (3) maximum of 3 samples (sequences) are included from a species. After processing, multiple alignments were made from the selected sequences using MEGA6 and sequence length (bp), conserved sites (%), variable sites (%), and GC content (%) of each data set were recorded.

#### Dataset 2 Amplicon profile of medicinal Acanthaceae species

Sequences of the most suitable markers evaluated from dataset 1 were searched and downloaded from GenBank using keyword ‘(medicinal species) + (region title).’ The search results were filtered with criteria similar to *dataset 1*. After processing, multiple alignments were made from the selected sequences using MEGA6 and sequence length (bp), conserved sites (%), variable sites (%), and GC content (%) of the data set were recorded.

### Plant materials and DNA isolation

Thai herb species in Acanthaceae were the main focus of the study. Dried plant tissues for DNA extraction were kindly provided by Queen Sirikit Botanic Garden (QSBG) (Table 1). The plant materials were ground with liquid nitrogen, and 100 mg of fine powder was then used for DNA extraction with the Nucleospin Plant II kit (Macherey-Nagel, Germany) following the manufacturer’s instruction. The DNA was stored at −20 °C for further use.

**Table 1:** List of medicinal plants species used in this study

Genus	No.	Species	Voucher Number	Genus-level discrimination test	Species-level discrimination test
*Acanthus*	1.	*Acanthus ebracteatus* Vahl	QBG 2933		•
	2.	*Acanthus montanus* T. Anderson	QBG 33204	•	•
*Andrographis*	3.	*Andrographis paniculata* (Burm.f.) Wall.ex Nees	QBG 65952	•	•
*Barleria*	4.	*Barleria cristata* L.	QBG 66149		•
	5.	*Barleria lupulina* Lindl.	QBG 39106	•	•
	6.	*Barleria strigosa* Wild.	QBG 65866		•
*Clinacanthus*	7.	*Clinacanthus nutans* (Burm.f.) Lindau	QBG 39490		•
	8.	*Clinacanthus siamensis* Bremek.	QBG 66156	•	•
*Hemigraphis*	9.	*Hemigraphis alternata* T. Anderson	QBG 41821	•	•
*Justicia*	10.	*Justicia adhatoda* L.	QBG 33096	•	•
*Rhinacanthus*	11.	*Rhinacanthus calcaratus* Nees	QBG 51778	•	•
	12.	*Rhinacanthus nasutus* Kuntze	QBG 63282		•
*Thunbergia*	13.	*Thunbergia fragrans* Roxb.	QBG 69714		•
	14.	*Thunbergia grandiflora* (Roxb. Ex Rottler) Roxb.	QBG 36867		•
	15.	*Thunbergia laurifolia* Lindl.	QBG 65418		•
	16.	*Thunbergia similis* Craib	QBG 21109	•	•

### High resolution melting (HRM) analysis

#### Genus-level discrimination

Eight samples from different genera were arbitrarily selected to test the feasibility of Bar-HRM in genus-level discrimination. To determine the characteristic melting temperature (T_m_) for each sample that could be used to distinguish among the different species, DNA amplification using real-time PCR and DNA was performed using the Eco Real-Time PCR system (Illumina, San Diego, USA). The reaction mixture for the real-time PCR and HRM analysis consisted of a total volume of 10 μl, containing 5 μl of MeltDoctor HRM Master Mix (Applied Biosystems, USA), 0.2 μl of 10 mM forward primer, 0.2 μl of 10 mM reverse primer, 1 μl of 25 ng DNA and 3.6 μl of ddH_2_O. The primer pair was derived from the ITS2 sequence data retrieved from an online database (GenBank) (Forward 5′- CGCCTGCTTGGGCGTCATGGC -3′ and Reverse 5′- GGGCCTCGCCTGACTTGGGGCC -3′). Fluorescence dye was used to monitor both the accumulation of the amplified product and the high-resolution melting process in order to derive the T_m_ value during PCR.

The thermocycling reactions (PCR) were conducted in a 48-well Helixis plate using an initial denaturing step of 95 °C for 5 min followed by 35 cycles of 95 °C for 30 s, 57 °C for 30 s, and 72 °C for 20 s. The fluorescent data were acquired at the end of each extension step during the PCR. Before HRM, the products were denatured at 95 °C for 15 s, and then annealed at 50 °C for 15 s to randomly form DNA duplexes. For the HRM experiments, fluorescence data were collected every 0.1 °C. EcoStudy software (version 5.0) was used to analyse the T_m_. The negative derivative of the fluorescence (F) over temperature (T) (dF/dT) curve displays the T_m_, and the normalised raw curve depicts the decreasing fluorescence vs. increasing temperature. To generate normalised melting curves and differential melting curves, pre- and post-melt normalization regions were set to define the temperature boundaries of the normalised and difference plots.

##### Species-level discrimination

To further investigate the feasibility of Bar-HRM in species-level discrimination, all 16 samples were included (Table 1). The protocol carried out was similar to the genus-level discrimination test.

## Results

### In silico analyses

#### Sequence profile of DNA barcodes of plants in family Acanthaceae (Dataset 1)

The number of sequences downloaded from GenBank of ITS2, *matK*, *trnH-psbA spacer region*, *rbcL* and *trnL* were 606, 222, 287, 258 and 770 respectively ranging between 225 to 895 bp for ITS2, 535 to 2498 bp for *matK*, 223 to 580 bp for *trnH-psbA spacer region*, 399 to 1461 bp for *rbcL* and 113 to 948 bp for *trnL* (Table 2). *Dataset 1* was constructed after filtration of irrelevant, unverified, ambiguous and outnumbered sequences followed by multiple alignment. The number of remaining sequences of ITS2, *matK*, *trnH-psbA spacer region*, *rbcL* and *trnL* were 399, 100, 91, 145 and 189 covering 313, 58, 84, 96 and 176 species from 48, 24, 10, 47 and 85 genera with the sequence lengths of 379, 570, 587, 457 and 601 bp respectively (Table 2).

**Table 2:** Search results of five DNA regions of Acantaceae species retrieved from GenBank

Regions	Number of		Length (bp)	
	Sequences	Genera	Min	Max
*matK*	222	25	535	2498
*psbA-trnH*	287	55	223	580
*rbcL*	258	47	399	1461
*trnL*	770	112	113	948
ITS2	606	115	225	895

Across all search results from GenBank, the most abundant retrieved sequences belonged to *trnL* followed by ITS2 > *trnH-psbA spacer region* > *rbcL* > *matK* respectively. The average length of *matK* was found to be the longest among five analysed regions (1280 bp). Additionally, as a result of sequence filtration with criteria mentioned above, ITS2 remained the largest group of samples covering highest number of species although *trnL* was observed to be broader in terms of genera. (Table 2). The analysis of dataset 1 indicated that ITS2 regions of plants in family Acanthaceae, apart from its shortest length, had the highest percentage of variable sites. Moreover, ITS2 was also on the top in terms of GC content among the five selected markers. While *trnH-psbA spacer region* possessed the second highest proportion of variable sites, it however had a considerably low GC content ranking it at the bottom. Furthermore, although *rbcL* exhibited second highest GC content, it was the least variable marker which contained the lowest percentage variable sites (Table 3). All in all, the in silico results suggested that ITS2 was most likely to be the most suitable marker for discrimination among Acanthaceae species.

**Table 3:** *Acanthaceae* sequences profile of five selected regions (*matK*, *psbA-trnH*, *rbcL*, *trnL* and ITS2) in *dataset 1 and 2*

	*dataset 1*			*dataset 2*		
Markers	*mat*K	*psbA-trnH*	*rbcL*	*trn*L	ITS2	ITS2
Sequences in analysis dataset	100	91	145	189	399	8
Genera	24	10	47	85	48	8
Species	58	85	96	176	313	8
Characters (bp)	570	587	457	601	379	211
Variable characters (%)	50.52	62.35	21.66	57.07	88.12	70.62
Average %GC content	34.04	27.10	45.65	35.73	66.79	70.11

#### Amplicon profile of medicinal plants in Acanthaceae (Dataset 2)

Two main criteria were considered in order to obtain successful results in the HRM analysis: (i) the primer pair should generate a PCR product not exceeding 300 bp, (ii) the primer pairs should cover enough variable sites to enable discrimination among the tested species. Here, we tested whether the most suitable markers from analysed *dataset 1*, ITS2, would still be suitable when amplified product was reduced to be less than 300 bp. The multiple alignment of retrieved sequences from GenBank in *dataset 2* were trimmed using ITS2 Bar-HRM primers which resulted in various size of generated amplicons depending on species as following *Acanthus spinosus* 186 bp, *Andrographis paniculata* 190 bp, *Clinacanthus nutans* 202 bp, *Hemigraphis alternata* 197 bp, *Justicia adhatoda* 203 bp, *Ruellia tuberosa* 203 bp and *Thunbergia alata* 208 bp.

Initially, we planned to use ITS2 sequence particularly from all of the medicinal species as same as our samples from QSBG (Table 1). However, the sequence data was not available for most of them. Thus, we relaxed our subject to randomly selected plant species under the same genus. As a result of ITS2 search of medicinal genera, 8 genera were found on GenBank database which incidentally matched 4 medicinal species available at QSBG. Multiple alignment of ITS2 amplicons from seven genera of medicinal species resulted in 211 bp sequences. The analysis of *dataset 2* indicated that the expected amplicons would contain lower variability (70.62%) but richer GC content (70.11%), compared to *dataset 1* in which non-medicinal species were also included (Table 3).

### Bar-HRM analyses

#### Genus-level discrimination test

**Fig. 3:**
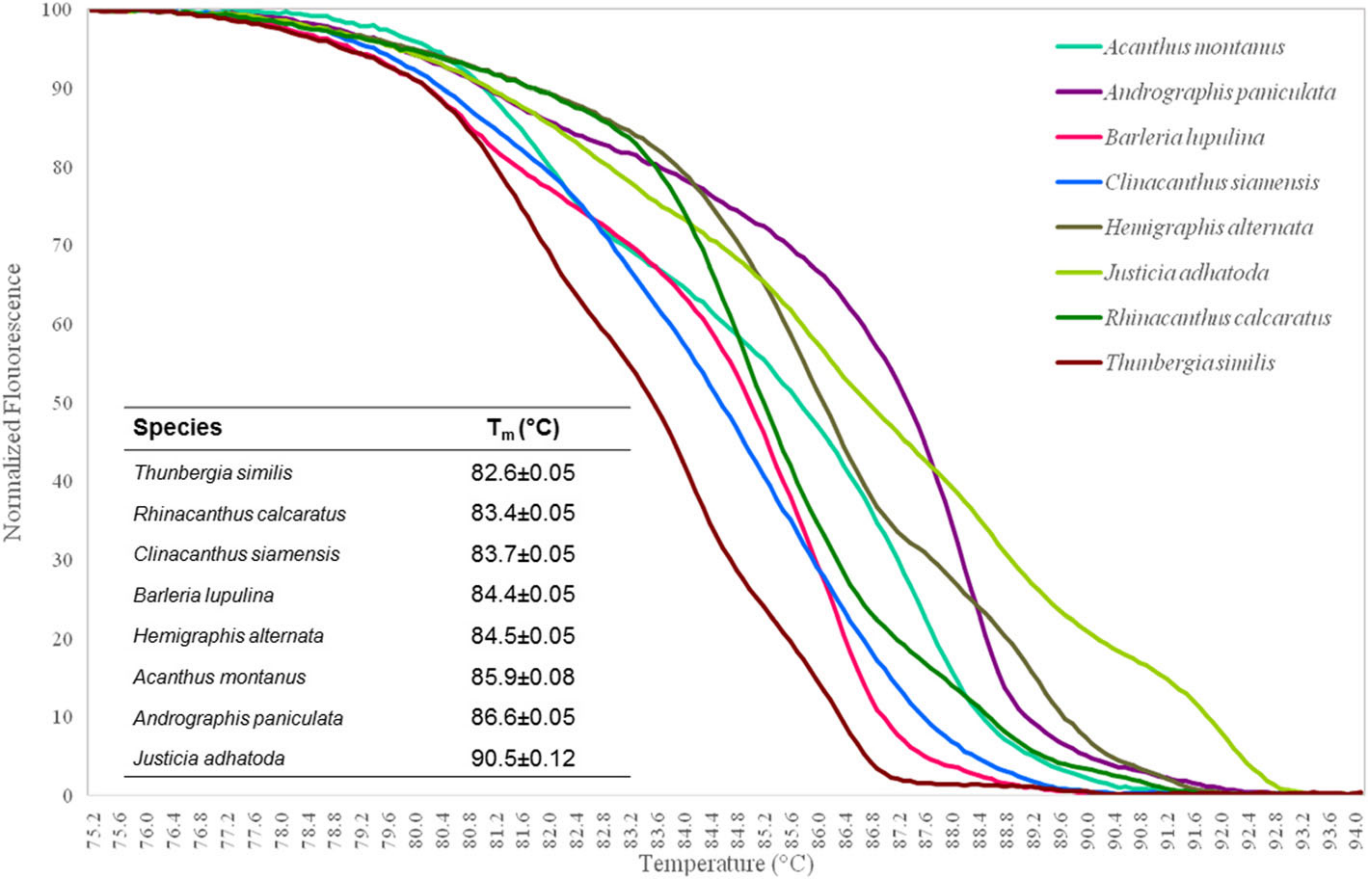
The normalised plot shows the differentiation of melting temperature (T_m_) of each ITS2 amplicon from each species, generated by high resolution melting (HRM) analysis. Eight Acanthaceae species were included

The feasibility in genus-level identification of Bar-HRM technique was examined with eight medicinal species from different genera in family Acanthaceae using ITS2 primers. HRM analysis was performed in triplicate on each of the eight tested species to establish the T_m_. The shapes of the melting curves were analysed using EcoStudy Software (version 5.0) to distinguish the different plant species. The melting temperatures of all tested samples were found ranged from 82.6–90.5 °C. It was observed that the melting temperatures of *Barleria lupulina* and *Hemigraphis alternata* were very close to one another (84.4–84.5 °C). Therefore, apart from melting temperature, we also need to use the shapes of melting curve in species identification. The melting profiles of all amplicons are illustrated in Fig. 3. The analysis is presented by means of conventional derivative plots, which show that the T_m_ value of each species is represented by a peak. The individual melting curves were reproducibly achieved from each sample. The samples of the eight different species could be easily distinguished using HRM analysis with ITS2 primer pairs.

#### Species-level differentiability test

As the genus-level discrimination test result suggested that ITS2 is a good primer candidate for identification plant species in Acanthaceae, we deepened our investigation into species-level by including more species in each genus (total of 16 species as indicated in Table 1) The ITS2 primers set was used for the amplification of DNA-fragments from all 16 samples, and the amplicons were analysed using HRM to define T_m_. Comparing T_m_ of species belonging to the same genus found that none of medicinal species under the same genus were shared or close to one another.

**Fig. 4:**
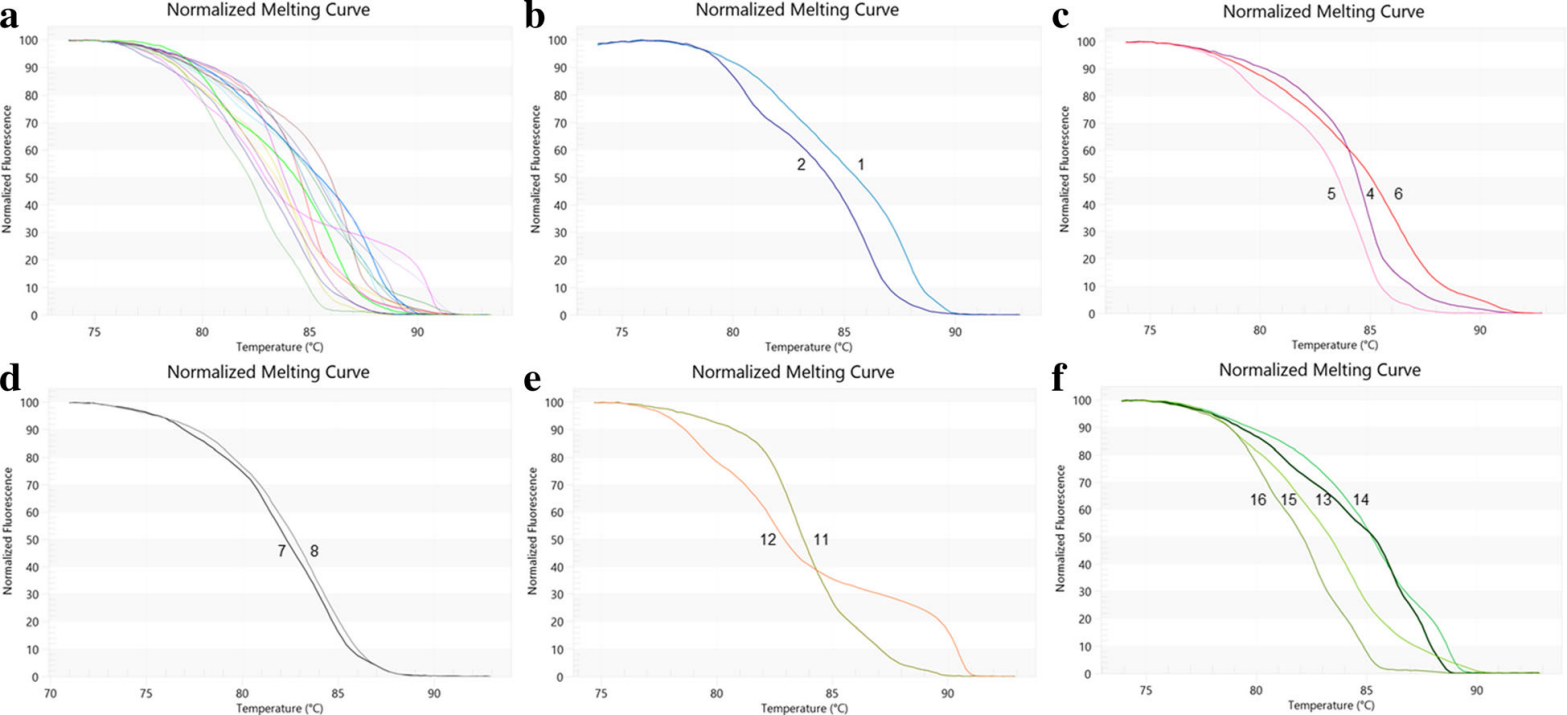
Melting profiles of 16 Acathaceae species generating from HRM analysis using ITS2 primers. Normalised curves of (**a**) all 16 species, (**b**) species from genus *Acanthus*, (**c**) species from genus *Barleria*, (**d**) species from genus *Clinacanthus*, (**e**) species from genus *Rhinacanthus*, and (**f**) species from genus *Thunbergia*

The melting profiles of all amplicons are illustrated in Fig. 4a. The analysis is presented by means of conventional derivative plots, which show that the T_m_ value of each species is represented by a peak. The melting profiles of all 16 tested species in Fig. 4b–f revealed that all species under the same genus could be easily distinguished except for those in the genus *Clinacanthus* (Fig 4d).

## Discussion

Material adulteration or substitution can be found in processed herbal products due to the indefinable structure. This could pose a serious problem in quality control in medicine production especially those with high consumption and economic value. Acanthaceae is a family that consists of a number of economically significant herbal species which are commonly found in commercialised forms [21, 24]. Quality control of herbal products should begin at the earliest stage of production therefore accurate species identification of raw materials is needed. DNA barcoding is a DNA-based method that has been proved successful in species identification regardless to the physical appearance of sample. In brief, DNA barcode is the use of a short standardised DNA region to identify organism to species level. In plants, a number of barcode regions, both from nuclear and plastid DNA, have been proposed yet none was found applicable for all land plants [33]. High Resolution Melting (HRM) is a technique that can be used to characterise the variants in DNA fragment. HRM involves the monitoring of decrease in fluorescent level emitted from fluorescent dye intercalating on double-stranded DNA as it is denatured by the gradually increasing temperature [15]. Variations in length, GC content and base sequence will alter the melting profile which is defined by a plot between temperature and fluorescent level [17]. HRM coupled with DNA barcode (called Bar-HRM) has been proved promising in species identification and detection of adulterants in a number of studies. However, all previous applications of Bar-HRM were the use of the technique to detect or indicate species in end product. Therefore here, we would like to evaluate the use of Bar-HRM to identify plant species at early stage of the production like raw material identification.

In our study, to address the most suitable marker for Bar-HRM of raw material plants in family Acanthaceae, the readily available online sequence database, GenBank, was retrieved for in silico analyses. In doing so, two datasets were generated. The *dataset 1* was constructed to compare each marker’s sequence profile including one marker from nuclear genome (ITS2) and four from plastid genome (*matK*, *rbcL*, *trnH-psbA spacer region*and *trnL*) of plant species in Acanthaceae. The DNA barcodes were chose based on several works on plant species identification which have been recently reported. The most suitable candidate from *dataset 1* was found to be the ITS2 which then selected to construct *dataset 2*. An in silico study from other group also demonstrated that ITS2 sequences could successfully identify 76.58% of plants across dicotyledons, monocotyledons, gymnosperms, ferns and mosses [34]. Moreover, according to experimental study focused only on Chinese medicinal plants, specie-level identification success rate of ITS2 was as high as 92.7% [35]. As a result of data mining from Genbank in our study, ITS2 was the second most abundant sequence after *trnL*. The high number of sequences indirectly reflected the level of research interest of plant DNA markers in family Acanthaceae. After filtering raw sequences using criteria as follows; verified sequence, species name given, containing less than 3% of ambiguous bases and maximum of three sequences per species, the number of remaining sequence was highest in ITS2. It was found that ITS2 covered the largest number of species although *trnL* and *rbcL* included broader genera. This demonstrated the biases of studying in a certain groups of plants in family Acanthaceae. The genus bias could be resulted by the popularity of ITS2 in phylogenetic construction and taxonomic classification [36, 37].

Bar-HRM primers were designed to give a smaller size of DNA product than the universal primers because a larger DNA size reduces the distinction between variants [38]. Here, we use tailored reduced amplicons from the ITS2 barcode region (211 bp) in combination with HRM to identify raw material medicinal plants in Acanthaceae. Not only the sequence length but also the nucleotide variation within sequences influence the dissociation energy of the base pairs and result in different T_m_ values in HRM analysis. Therefore the GC content and variable sites found within the amplicons were reported. The average GC content of expected amplicons generated by ITS2 primers of the analysed medicinal plants species was 70.11% and the 149 of 211 bp (70.62%) were found to be variable sites. Thus, these ITS2 primers, were predicted to perform well in HRM analyses with the medicinal plant species in question, based on the sequences extracted from GenBank.

Proposed to be a standard plant barcode, ITS2 is a non-coding nuclear DNA located between 5.8S rRNA (or 2.8 S rRNA in prokaryote) and 25S rRNA genes [6]. ITS2 is a part of ITS complex, although subsequent studies showed that only ITS2 sufficed discrimination power in various groups of plants [34]. ITS2 has strengths in high discriminatory power, variability and sequence quality [34, 35]. To be suitable for HRM, GC content should also be considered alongside with those three strengths which would enhance the sensitivity in species discrimination by HRM [17, 38]. To evaluate the ITS2 primers in medicinal plant species discrimination by Bar-HRM, the medicinal plant species in Acanthaceae using HRM analysis were performed. HRM analysis was performed in triplicate to establish the T_m_ of each species. The shapes of the melting curves were analysed to distinguish the different plant species. Bar-HRM species identification success rates were assessed for genus level as well as for species level to establish the success rate in medicinal plant species discrimination of the primer pair. Eight species belonging to eight genus were chosen for genus-level discrimination test and 16 species were included in the specie-level discrimination test. The melting profiles of each plant species obtained from both tests were visually distinguishable although the melting temperatures of some samples were close together. There appeared to be no relationship between the taxonomical relatedness and melting temperature (Fig 3).

Interestingly, the melting curves of *C. nutans* and *C. siamensis* are the only sample group in species-level test that could not be distinguished from each other (Fig 4d). Our further literature review on these two species revealed that *C. siamensis* is a synonym of *C. nutans* according to The Plant List [39]. Although they possess different Thai common names (*C. nutans* = ‘Pha-Ya-Yo’ or ‘Sa-Led-Pang-Porn-Tua-Mea’ meaning female mongoose’s saliva, *C. siamensis* = ‘Lin-Ngu-Hao’ or less common ‘Sa-Led-Pang-Porn-Tua-Poo’ meaning male mongoose’s saliva) and are recognized as two separated species by Queen Sirikit Botanic Garden. Here, Bar-HRM technique using ITS2 was unable to discriminate *C. nutans* from *C. siamensis* based on their DNA data. Although, morphological data based on the external form of organisms, have been and still are used most in classification, numerous recent works have proposed integration of different types of data e.g. morphological, molecular, anatomical and ecological data [32]. In our opinion, DNA as molecular data are among the most informative and conspicuous.

## Conclusion

The HRM analysis has a number of advantages (1) the HRM analysis method is highly sensitive detecting 1%–0.1% presence of adulterated sample; (2) it is a high throughput technique that is capable of analysing multiple samples at the same time; (3) no post PCR processes needed thus cross-contamination could be avoided; (4) the sample genotype can be evaluated directly based on HRM curve analysis; (5) no specific probes and sequencing required. All in all, HRM analysis is an economically effective method which can significantly simplify the procedure and shorten the time of analysis, although sequencing would be needed in some cases. We report that Bar-HRM technique using ITS2 primers was feasible in discrimination of the selected Thai medicinal plants in Acanthaceae and therefore, our developed technique could be used to ensure the safety and efficacy of Thai herbal product particularly those products containing tested species from Acanthaceae. Furthermore, the use of molecular data in aiding taxonomy and systematics seems to be an intriguing approach, particularly when morphological methods fail in delimiting species.

## Abbreviations

GACP: Good Agricultural and Collection Practice
GMP: Good Manufacturing Practice
HRM: High resolution melting
